# Sociodigital Determinants of eHealth Literacy and Related Impact on Health Outcomes and eHealth Use in Korean Older Adults: Community-Based Cross-Sectional Survey

**DOI:** 10.2196/56061

**Published:** 2024-08-13

**Authors:** Myat Yadana Kyaw, Myo Nyein Aung, Yuka Koyanagi, Saiyud Moolphate, Thin Nyein Nyein Aung, Hok Ka Carol Ma, Hocheol Lee, Hae-Kweun Nam, Eun Woo Nam, Motoyuki Yuasa

**Affiliations:** 1 Department of Global Health Research, Graduate School of Medicine, Juntendo University, Hongo-2-1-1 , Bunkyo Ku, Tokyo, 113-8421, Japan, 81 338133111 ext 2994, 81 338181168; 2 Faculty of International Liberal Arts, Juntendo University, Tokyo, Japan; 3 Advanced Research Institute for Health Sciences, Juntendo University, Tokyo, Japan; 4 Department of Judo Therapy, Faculty of Health Sciences, Tokyo Ariake University of Medical and Health Sciences, Tokyo, Japan; 5 Department of Public Health, Faculty of Science and Technology, Chiang Mai Rajabhat University, Chiang Mai, Thailand; 6 Department of Family Medicine, Faculty of Medicine, Chiang Mai University, Chiang Mai, Thailand; 7 S R Nathan School of Human Development, Singapore University of Social Sciences, Singapore, Singapore; 8 Department of Health Administration, Yonsei University Graduate School, Wonju, Republic of Korea; 9 Department of Preventive Medicine, Wonju College of Medicine Yonsei University, Wonju, Republic of Korea; 10 Department of Health Administration, Software Digital Healthcare Convergence College, Yonsei University, Wonju, Republic of Korea

**Keywords:** eHealth literacy, eHEALS, electronic health information, eHealth information, health literacy, health education, eHealth education, health training, eHealth training, digital health, digital technology, digital intervention, digital interventions, gray digital divide, healthy aging, gerontology, geriatric, geriatrics, older adult, older adults, older person, older people, aging, aging, eHealth Literacy Scale

## Abstract

**Background:**

eHealth literacy is an essential skill for pursuing electronic health information, particularly for older people whose health needs increase with age. South Korea is now at the intersection of a rapidly digitalizing society and an increasingly aged population. eHealth literacy enables older people to maximize the effective use of emerging digital technology for their health and quality of life. Understanding the eHealth literacy of Korean older adults is critical to eliminating the gray digital divide and inequity in health information access.

**Objective:**

This study aims to investigate factors influencing eHealth literacy in older Korean adults and its impact on health outcomes and eHealth use.

**Methods:**

This was a cross-sectional survey. Community-dwelling older adults 65 years and older in 2 urban cities in South Korea were included. eHealth literacy was measured by the eHealth Literacy Scale. Ordinal logistic regression was used to analyze factors associated with eHealth literacy and multivariate ANOVA for the impact of eHealth literacy on health outcomes and eHealth use.

**Results:**

In total, 434 participants were analyzed. A total of 22.3% (97/434) of participants had high eHealth literacy skills. Increasing age, higher monthly income, and time spent on the internet were significantly associated with eHealth literacy (*P*<.001), and social media users were 3.97 times (adjusted odds ratio 3.97, 95% CI 1.02‐15.43; *P*=.04) more likely to have higher skill. Higher eHealth literacy was associated with better self-perceived health and frequent use of digital technologies for accessing health and care services (*P*<.001).

**Conclusions:**

Disparity in socioeconomic status and engagement on the internet and social media can result in different levels of eHealth literacy skills, which can have consequential impacts on health outcomes and eHealth use. Tailored eHealth interventions, grounded on the social and digital determinants of eHealth literacy, could facilitate eHealth information access among older adults and foster a digitally inclusive healthy aging community.

## Introduction

Declining fertility rates and longer life expectancy primarily contribute to the demographic transition into an aging population worldwide. This transition is more prominent in Asia, where Japan, South Korea, China, and Singapore entered the aged society within a period of 20 years. In 2021, South Korea hosted 8.71 million people aged 65 years and older (16.8% of the total population), and the proportion increased by 5.1% compared to 2020 [1]. The rapid increase in the older population underscores the importance of an age-friendly environment in which older people can think or do what they value and age in a place to which they are attached.

Nowadays, with technological integration in every aspect of life, digital inclusiveness has become a basic component of an age-friendly environment. Traditional health promotion and long-term care systems could be upgraded into more productive and efficient systems with the diffusion of technology and digital devices. Gerontechnology, an emerging scientific field applying technological advances in the aging sector, is now used to improve health and social connections among older adults [2]. It is more evident in the COVID-19 pandemic, where physical distancing was inevitable. Digital health promotion activities and exercise videos were used to maintain over 20-year-old community exercise programs in Japan and preserve older people’s functional mobility [3]. The application of robotic technology for social connections of residents also showed better engagement and positive user perception in long-term care facilities compared to conventional methods such as telephone communication [4]. Additionally, older adults have a positive attitude toward using digital technology and are willing to use it for health purposes and to maintain activities of daily living [5,6].

Although older people have a positive attitude and are willing to use digital health interventions and devices, they face challenges and limitations when adopting digital technology. First, having access to digital devices and networks is the initial step in adopting digital technology and its associated interventions [7]. Aged countries, such as Japan, South Korea, and Singapore, prioritized digitalization and promoted and implemented policies for digital inclusiveness [8]. Due to government efforts, the gap in access to digital devices and the internet has narrowed. In 2022, the digitization level reached 76.2% among South Korea’s vulnerable populations (older people, low-income individuals, people with disabilities, fishermen, and farmers), and digital access was as high as 96% of the total population [9]. Regarding older people, 69.9% had access to digital information in 2022, increasing from 69.1% in 2021 [9]. Ownership of digital devices and access to the internet do not guarantee the actual application of digital technology for health. Another barrier for older adults to fully embrace the benefits of technology is not knowing how to acquire and use information obtained in the digital context to make effective health decisions [10].

According to Norman and Skinner [11], eHealth literacy is defined as “the ability to seek, discover, evaluate and appraise eHealth information and apply the acquired knowledge to solve health problems.” Older adults often see eHealth literacy as a barrier to successfully using electronically delivered health information and interventions [10]. Due to technological advancements, health information is one-click away, and older adults are faced with information overload, in which misleading information could be exposed physically and digitally. On the other hand, internet use for health-related searches by older American adults increased from 24.8% in 2009 to 43.9% in 2018 [12]. Due to the prevalence of chronic diseases and age-related disorders in older age, there are pros of searching health information on the web. However, older adults could be harmed by fake information on the web if they lack adequate skills to identify the correct source and facts. Therefore, being eHealth literate is essential, and exploring the factors associated with older adults’ eHealth literacy is crucial.

Extensive research has shown that age, sex, level of education, marital status, economic conditions, and social support contribute to the eHealth literacy of older adults [13]. Arcury et al [14] stated that internet use is positively associated with eHealth literacy. Little published data exist on the relationship between social media and eHealth literacy, especially in older populations. In addition, having a good command of eHealth literacy skills enhances the physical, mental, and social well-being of older people [15]. Higher eHealth literacy skill is a protective factor against cognitive decline and is positively associated with health-promoting behaviors and better health outcomes [16,17]. There have been few attempts to investigate the impact of eHealth literacy on perceived health status and the use of digital devices and the internet for health, particularly in the older population. The need for scientific literature in this field is more urgent in South Korea, which is becoming a super-aged society, and the proportion of older adults is expected to be as high as 44% in 2050 [18]. Additionally, the Ministry of Health and Welfare in South Korea started a pilot project to use artificial intelligence and the Internet of Things to improve the health care of older adults [19]. Advanced technology is readily diffused into the daily lives of older Korean adults, and knowledge of embracing eHealth information becomes inevitable. Therefore, this study aims to fill the gap by exploring the factors associated with eHealth literacy in community-dwelling older Korean adults and the impact of eHealth literacy on health-related outcomes and digital technology use for health purposes.

## Methods

### Participants, Setting, and Data Collection

It is a community-based survey conducted in 2 urban cities of the Republic of Korea, Wonju-si and Yeoju-si, in 2022. This cross-sectional study is part of the digitally inclusive healthy aging communities study, a cross-cultural study in 4 rapid aging countries: Japan, the Republic of Korea, Singapore, and Thailand [20]. After obtaining ethics approval, participation in the study was announced in the study area. It was accomplished through the cooperation of local senior welfare centers and senior citizen centers. Community-dwelling older adults aged 65 years and older, both male and female adults with ongoing health promotion activities in residing communities, were included in the study.

The sample size was calculated by 1 sample estimation of proportion in Stata SE (version 16.0; Stata Corp) based on the proportion of internet use among older adults in South Korea. The calculated sample size was inflated by 20% for nonresponses. A sample of 444 participants responded to the questionnaire, and 434 were analyzed after excluding 10 participants who were younger than 65 years.

### Ethical Considerations

The Juntendo University Ethical Committee (approval E22-0057-M01) and Yonsei University Institutional Review Board (approval 1041849‐202304SB-073-02) approved the ethics of the digitally inclusive healthy aging communities study. Participation in the study was completely voluntary. The study’s purposes and procedures were thoroughly explained, and written informed consent was obtained. Data were collected using a structured questionnaire in the participants’ native language (Korean). The data were anonymized and identifiable features were not included. The participants were compensated with a financial incentive equivalent to US $10 for answering the questionnaire.

### Measures

#### Demographic Characteristics

Demographic characteristics of age, sex, education, living arrangements, and financial status were investigated. Age was described as a continuous variable for distribution and divided into 4 categories (65-70, 71-75, 76-80, and >80 years) for eHealth distribution and regression analysis. Education was asked for the highest level of completed education, with 4 groups (did not go to school, primary school completed, junior high school completed, and high school and above). For living arrangements, the participants were asked whether they lived alone or with someone and categorized into living alone, living only with a spouse, and living only with children or grandchildren. Financial status included average income per month based on the 4 income quartiles in South Korea, which is divided into 2 groups of low (less than or equal to 1 million Won; a currency exchange rate of ₩ 1=US $0.0008 is applicable) and high (more than 1 million Won) and a dichotomous question on receiving a pension of any type.

#### Internet Use and Social Media Use

We determined internet use by a dichotomous question about the internet use derived from the internet environment and digital devices used to access the internet. The frequency is then determined by number of hours per day and number of days per week spent on the internet. Negative responses in the dichotomous question, 0 hours and 0 days of using the internet, were defined as internet nonusers. Positive responses in the dichotomous question, more than 0 hours and 0 days of using the internet, were defined as internet users. Engagement in 1 or more social media platforms prevalent in South Korea determined social media use.

#### Health-Related Outcomes and eHealth Use

Self-perceived health status was measured using a single-item 4-point Likert scale (1=very healthy to 4=not healthy at all). A single-item 4-point scale has been used to reflect the subjective health status of community-dwelling older adults in longitudinal and cross-sectional studies and to predict mortality, health outcomes, and digital use among older adults [21-24]. Annual medical checkup was measured using a 5-point Likert scale ranging from 1=never to 5=always, and the participants were asked how frequently they participated in regular medical checkups. The technology used in health was measured by how often participants used the internet and digital technology to improve eating habits and access health care and long-term care services. The scale was a 5-point Likert scale ranging from 1=never to 5=always. For analysis, annual medical checkups, digital technology, and internet use for health purposes were regrouped according to their participation and frequency of use as never or nonuser, low participation or low user, and frequent participation or frequent user.

#### eHealth Literacy

eHealth literacy was measured by an 8-item 5-pointed eHealth Literacy Scale (eHEALS) developed by Norman and Skinner [25]. The scale was developed using the Lily model of eHealth literacy, which consisted of 6 aspects (healthy, traditional, information, scientific, media, and computer literacy). The scale tends to measure perceived skill and comfort with eHealth rather than the actual skill itself [25]. Although eHEALS was primarily developed for using computers for health purposes, it has been validated with the use of mobile devices and social media and shows good reliability [26,27]. In this study, the scale has a Cronbach α reliability coefficient of 0.99, which indicates high internal consistency and reliability. For distribution, eHealth literacy is categorized into 3 groups: lack of eHealth literacy (eHEALS 8‐15.9), low to moderate eHealth literacy (eHEALS 16‐31.9), and high eHealth literacy (eHEALS 32‐40) [28].

### Statistical Analysis

The statistical analysis was done by using Stata SE (version 16.0; StataCorp). Sociodemographic characteristics, the use of the internet and social media, eHealth literacy, self-perceived health, annual medical checkups and eHealth use for improving eating habits, and access to health care services and long-term care services were described by descriptive statistics. Frequency and percentage were used to describe categorical data and mean and SD for continuous data. The normality of the data was checked by using the Shapiro-Wilk test. The distribution of eHealth literacy across sociodemographic factors, internet, and social media use were described by descriptive statistics. A Kruskal Wallis H test and Mann-Whitney *U* tests analyzed the difference in mean scores.

Ordinal logistic regression was used to identify factors influencing eHealth literacy. Univariate ordinal logistic regression treated eHealth literacy as the dependent variable and age, sex, education, monthly income, pension receiving status, living arrangement, internet use, and social media use as independent variables. The statistically significant variables (*P*<.2) and conceptually relevant variables were included in the multivariate analysis. Association was reported as an adjusted odds ratio (aOR) and 95% CI. Statistical significance is defined as a *P* value ≤.05 with a 95% CI. Age, sex, income, and education were included as covariates in the multivariate analysis.

Furthermore, multivariate analysis of variance was applied to assess the difference in health-related outcomes such as perceived health status, annual medical checkup, and eHealth use to improve eating habits, access to health care, and long-term care services among the different orders of eHealth literacy. The covariates for the impact of eHealth literacy on health outcomes and eHealth use were age and sex.

## Results

### Overview

The mean age was 76.8 (SD 6.6) years, and participants 80 years and older occupied 30% (131/432) of the total sample. Of the total sample (N=434), 315 (72.6%) were female, and 192 (44%) participants of the sample had primary school education or lower. A total of 136 (31.5%) older people lived alone, 341 (78.6%) had monthly income lower than or equal to 1 million Won, and 385 (88.7%) received a pension (Table 1). Regarding digital technology use (N=434), 208 (47.9%) participants used the internet, and 184 (42.4%) engaged in social media. The mean days of internet use in a week were 2.4 (SD 2.9), and the mean hours of internet use in a day were 0.8 (SD 1.4). Over one-third of the sample population spent more than 3 days a week (142/404, 35.2%) and 1‐2 hours a day (155/404, 38.4%) on the web. The mean of the eHEALS score was 15.4 (SD 10.8). Over half of the participants (289/434, 66.6%) lacked eHealth literacy, and 97 (22.4%) had high eHealth literacy (Table 2).

Regarding health-related outcomes, almost every participant had an annual medical checkup (n=423, 97.9%). Over half of the participants (n=242, 55.8%) reported not being in good health. Regarding eHealth use, 353 (81.3%) participants did not have experience using digital technology to improve eating habits. A total of 350 (80.6%) and 370 (85.3%) users did not use digital technology to access health care services or long-term care services, respectively (Table 2).

**Figure 1.**
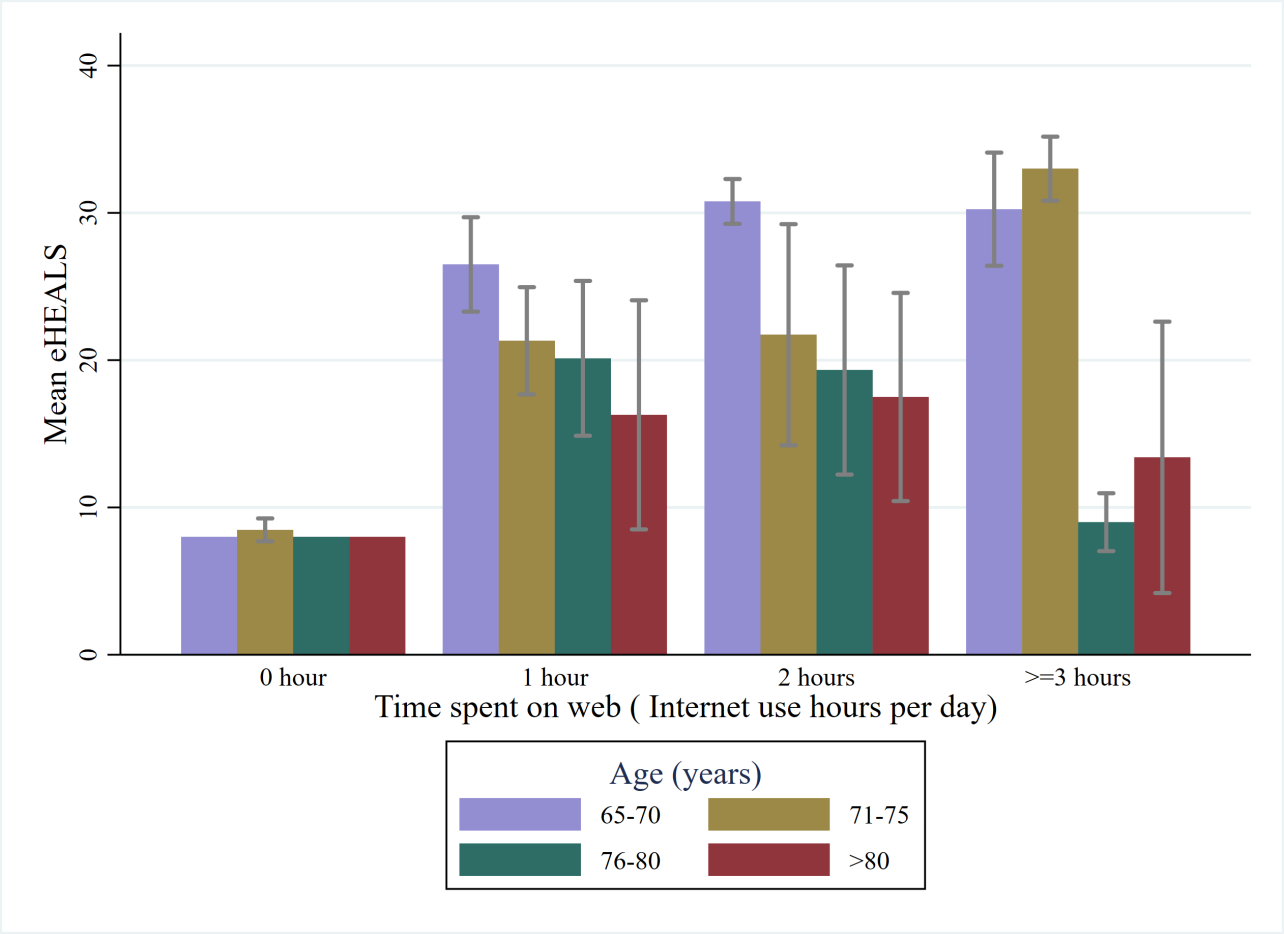
Level of eHealth literacy of different age groups by different categories of daily time spent on the web among older Korean adults. eHEALS: eHealth Literacy Scale.

Table 3 shows the distribution of eHealth literacy across different groups of sociodemographic factors and digital technology use. The results showed significant differences in mean eHEALS scores across all variables except for receiving pension. The level of eHealth literacy descends with older age and ascends with daily time spent on the web (Figure 1).

**Table 1.** Sociodemographic characteristics and digital technology use in older Korean adults (N=434).

		Values, n (%)	Mean (SD)
**Age (years**)			76.8 (6.6)
	65‐70	94 (22)	
	71‐75	108 (25)	
	76‐80	99 (23)	
	>80	131 (30.3)	
**Sex**			—^a^
	Male	119 (27.4)	
	Female	315 (72.6)	
**Education**			—
	Did not go to school	70 (16)	
	Primary school	124 (28.6)	
	Secondary school	93 (21)	
	High school and above	147 (33.8)	
**Living arrangements**			—
	Living alone	136 (31.5)	
	Living with spouse	241 (55.8)	
	Living with a child or grandchild	55 (13)	
**Monthly income^b^**			—
	Less than or equal to 1 million Won	341 (78.6)	
	More than 1 million Won	93 (21)	
**Pension receiving status**			—
	Not receiving	49 (11)	
	Receiving pension	385 (88.7)	
**Internet use**			—
	Nonuser	226 (52.1)	
	User	208 (47.9)	
**Days of internet use per week**			2.4 (2.9)
	0 days	223 (55.2)	
	1 to 3 days	39 (10)	
	More than 3 days	142 (35.2)	
**Hours of internet use per day**			0.8 (1.4)
	0 hours	219 (54.2)	
	1 hour	92 (23)	
	2 hours	63 (15)	
	3 hours and above	30 (7)	
**Social media use**			—
	Nonuser	250 (57.6)	
	User	184 (42.4)	

a Not applicable.

b A currency exchange rate of ₩ 1=US $0.0008 is applicable.

**Table 2.** eHealth literacy, health-related outcomes, and eHealth use distribution in older Korean adults (N=434).

		Values, n (%)	Mean (SD)
**eHealth Literacy Scale**			15.4 (10.8)
	Lack of eHealth literacy (8‐15.9)	289 (66.6)	
	Low to moderate eHealth literacy (16‐31.9)	48 (11.1)	
	High eHealth literacy (32-40)	97 (22.3)	
**Perceived health status**			—^a^
	Very healthy	65 (15)	
	Moderately healthy	127 (29.2)	
	Not very healthy	150 (34.6)	
	Unhealthy	92 (21.2)	
**Annual medical checkup**			—
	Never	9 (2.1)	
	Low participation	36 (8.3)	
	Frequent participation	389 (89.6)	
**Digital technology and the internet use to improve eating habits**			—
	Nonuser	353 (81.3)	
	Low user	53 (12.2)	
	Frequent user	28 (6.5)	
**Digital technology and the internet use to access health care**			—
	Nonuser	350 (80.6)	
	Low user	55 (12.7)	
	Frequent user	29 (6.7)	
**Digital technology and the internet use to access long-term care services**			—
	Nonuser	370 (85.3)	
	Low user	53 (12.2)	
	Frequent user	11 (2.5)	

a Not applicable.

**Table 3.** eHealth literacy distribution across sociodemographic factors, internet use and social media use in older Korean adults (N=434).

		eHealth Literacy Scale score, mean (SD)	*P* value
**Age (years)**			<.001
	65‐70	26.9 (9.7)	
	71‐75	16.6 (11.3)	
	76‐80	11.8 (8.3)	
	>80	9.2(4.5)	
**Sex**			.002
	Male	18.1 (11.7)	
	Female	14.5 (10.4)	
**Education**			<.001
	Did not go to school	11.2 (7.7)	
	Primary school	10.4 (6.7)	
	Secondary school	13.6 (9.8)	
	High school and above	23.0 (11.5)	
**Living arrangement**			.002
	Living alone	12.8 (9.1)	
	Living only with spouse	17.0 (11.6)	
	Living only with child or grandchild	14.6 (10.3)	
**Monthly income^a^**			<.001
	Less than or equal to 1 million Won	14.3 (10.2)	
	More than 1 million Won	19.8 (12.1)	
**Pension receiving status**			.97
	Yes	15.5 (10.9)	
	No	15.2 (10.2)	
**Internet use**			<.001
	Nonuser	8.3 (2.7)	
	User	23.2 (11.1)	
**Days of internet use per week**			<.001
	0 days	8.3 (2.4)	
	1 to 3 days	23.2 (9.6)	
	More than 3 days	24.7 (10.8)	
**Hours of internet use per day**			<.001
	0 hours	8.1 (1.1)	
	1 hour	22.6 (10.7)	
	2 hours	26.1 (9.9)	
	3 hours and above	25.4 (11.2)	
**Social media use**			<.001
	Nonuser	8.6 (3.2)	
	User	24.9 (10.6)	

a A currency exchange rate of ₩ 1=US $0.0008 is applicable.

### Social and Digital Determinants of eHealth Literacy

Ordinal logistic regression predicted eHealth literacy from sociodemographic factors and the use of digital technology. eHealth literacy was associated with age, income, internet use, and social media use, while other variables were controlled. Compared to the 65‐ to 70-year age group, the odds of having high eHealth literacy were decreased by 76% (aOR 0.24, 95% CI 0.1‐0.6) for the 76‐ to 80-year age group and 88% (aOR 0.12, 95% CI 0.03‐0.42) for the >80-year age group. A monthly income of more than 1 million Won per month increased the odds of having a higher eHealth literacy 2.8 times (aOR 2.8, 95% CI 1.35‐5.8) than a monthly income of less than or equal to 1 million Won. Univariate analysis showed that internet users had significantly higher eHealth literacy (odds ratio 108.57, 95% CI 42.63‐276.54; *P*<.001). In multivariable analysis, hours spent on the web daily were significantly associated with eHealth literacy. Users who spent an hour per day had 19.2 times (aOR 19.2, 95% CI 2.33‐158.81; *P*=.006), 2 hours per day had 25.7 times (aOR 25.72, 95% CI 2.83‐233.47; *P*=.004), and 3 hours and more had 35.3 times (aOR 35.39, 95% CI 3.70‐338.63; *P*=.002) higher eHealth literacy than nonusers of the internet. Social media users had 3.9 times (aOR 3.97, 95% CI 1.02‐15.43; *P*=.04) higher eHealth literacy than nonusers (Table 4).

**Table 4.** Univariate and multivariable analysis of factors associated with eHealth literacy in older Korean adults (n=400)^a^.

Independent variables		Univariate analysis, OR^b^ (95% CI)	Multivariable analysis, aOR^c^ (95% CI)
**Age (65‐70 as reference**)			
	71‐75	0.18^d^ (0.11‐0.32)	0.53 (0.24‐1.17)
	76‐80	0.06^d^ (0.03‐0.12)	0.24^f^ (0.09‐0.59)
	>80	0.02^d^ (0.009‐0.04)	0.12^f^ (0.03‐0.42)
**Sex (male as reference**)			
	Female	0.52^d^ (0.34‐0.78)	0.61 (0.32‐1.18)
**Education (did not go to school as reference**)			
	Primary school	0.78 (0.34‐1.79)	0.39 (0.09‐1.60)
	Secondary	2.01 (0.91‐4.41)	0.99 (0.26‐3.76)
	High school and above	11.12^d^ (5.44‐22.73)	1.46 (0.39‐5.47)
**Income (less than or equal to 1 million Won as reference**)^e^			
	More than 1 million Won	2.66^d^ (1.70‐4.16)	2.8^f^ (1.35‐5.81)
**Pension receiving status (receiving pensions as reference**)			
	No	1.01 (0.56‐1.82)	—^g^
**Living status (living alone as reference**)			
	Living with spouse	2.28^d^ (1.44‐3.61)	1.22 (0.61‐2.44)
	Living with child and grandchild	1.49 (0.77‐2.93)	1.02 (0.35‐2.91)
**Internet use (nonuser as reference**)			
	User	108.57^d^ (42.63‐276.54)	7.3 (0.59‐89.67)
**Days of internet use per week (0 days as reference**)			
	1 to 3 days	108.4^d^ (37.99‐309.31)	1.52 (0.26‐8.74)
	More than 3 days	157.93^d^ (60.25‐413.95)	1.45 (0.26‐7.94)
**Hours of internet use per day (0 hours as reference**)			
	1 hour	251.88^d^ (59.01‐1075.19)	19.23^f^ (2.33‐158.81)
	2 hours	530.87^d^ (119.98‐2348.9)	25.72^f^ (2.83‐233.47)
	3 hours and above	589.94^d^ (120.99‐2876.56)	35.39^f^ (3.7‐338.63)
**Social media use (nonuser as reference**)			
	User	87.34^d^ (42.56‐179.26)	3.97^h^ (1.02‐15.43)

a Model parameter: likelihood ratio (LR) *χ*2_17_= 390.6 (*P*<.001), pseudo *r*2=0.35, LR test of proportionality of odds=199.07 (*P*=.78).

b OR: odds ratio.

c aOR: adjusted odds ratio.

d *P*<2.

e A currency exchange rate of ₩ 1=US $0.0008 is applicable.

f *P*<.01.

g Not applicable.

h *P*<.05.

### Impacts of eHealth Literacy on Health-Related Outcomes and eHealth Use

Self-perceived health was significantly better in participants with higher mean eHealth literacy scores (very healthy groups: mean 22.2, SD 12.0 and unhealthy groups: mean 11.6, SD 8.1; *P*<.001). Participants with higher mean eHealth literacy scores attended annual medical checkups frequently. Regarding the use of digital technology for health, frequent users had a statistically significant higher mean in all 3 measures: the use of the internet and digital technology for improving eating habits (mean 25.4, SD 11.8; *P*<.001), access to health care services (mean 28.4, SD 10.4; *P*<.001), and long-term care services (mean 23.3, SD 14.1; *P*=.004; Table 5).

The influence of eHealth literacy on health-related outcomes was analyzed using multivariate ANOVA (Table 6). The result showed eHealth literacy had an effect at the multivariate level on self-perceived health status, digital technology, and internet use to improve eating habits to access health care and long-term care (*P*<.001). In multivariate analysis, the eHealth literacy level did not significantly influence participation in annual medical checkups.

**Table 5.** eHealth Literacy Scale (eHEALS) and different levels of health outcomes and digital technology use for health in older Korean adults (N=434).

Health outcomes and eHealth use		eHEALS score, mean (SD)	*P* value
**Perceived health status**			<.001
	Very healthy	22.2 (12.0)	
	Moderately healthy	18.8 (11.6)	
	Not very healthy	12.1 (8.8)	
	Unhealthy	11.6 (8.1)	
**Annual medical checkup**			.004
	Never	9.9 (4.4)	
	Low participation	10.0 (6.0)	
	Frequent participation	16.1 (11.1)	
**Use of digital technology and the internet to improve eating habits**			<.001
	Nonuser	14.3 (10.4)	
	Low user	17.9 (10.6)	
	Frequent user	25.4 (11.8)	
**Use of digital technology and the internet to access health care**			<.001
	Nonuser	14.3 (10.4)	
	Low user	16.4 (9.6)	
	Frequent user	28.4 (10.4)	
**Use of digital technology and the internet to access long-term care**			.004
	Nonuser	14.8 (10.6)	
	Low user	18.4 (10.9)	
	Frequent user	23.1 (14.1)	

**Table 6.** The influence of eHealth literacy on health outcomes and eHealth use in older Korean adults (n=430).

	Self-perceived health status	Annual medical checkup	Digital technology use to improve eating habits	Digital technology use to access health care services	Digital technology use to access Long-term care services
eHealth literacy (β)	−0.018^a^	0.001	0.016^a^	0.019^a^	0.008^a^
95% CI	−0.027 to −0.008	−0.003 to 0.004	0.01 to 0.02	0.013 to 0.025	0.004 to 0.013

a *P*<.001.

## Discussion

### Principal Findings

Competent in eHealth literacy skills is a characteristic of older people with healthy behaviors and those with better health knowledge and attitudes [15]. This study fills the gaps in eHealth literacy evidence by identifying social and digital determinants of eHealth literacy in community-dwelling Korean older adults and its consequential impact on subjective health status and digital technology application for lifestyle improvement and health needs.

In this study, older age, monthly income of lower or equal to 1 million Won, internet nonusers, and those not engaged in social media have a higher risk of having poor eHealth literacy (Table 4). Furthermore, the findings indicated that eHealth literacy has a significant effect on self-perceived health and eHealth use. It was also associated with lifestyle behaviors using digital resources for promoting health (Tables5  6), such as improving healthy eating habits. Using smartwatches in physical activity promotion is an ideal recent example globally [29]. Additionally, eHealth literacy enabled older persons to seek health care services digitally and access long-term care services digitally (Tables5  6). Integrating all these findings, we can highlight the significant influence of eHealth literacy upon general well-being, self-care, and opportunity to use health and social care services and health promotion through multiple evidence. The findings in other recent publications agreed with our findings [30,31].

The eHealth literacy of community-dwelling older Korean adults is relatively low, and 66.6% (n=289) need support and facilitation to improve their skills (Table 2). Since eHealth literacy is a predictor of eHealth information seeking, the disparity could result in inequity in health information access. Incompetency in eHealth literacy skills in older adults reflects lower self-efficacy in the effective use of electronically delivered information and services and the risk of exclusion from these services [10]. Therefore, regarding the country’s high-paced demographic and digital transition, urgent interventions in eHealth literacy promotion targeting high-risk groups should be prioritized to ensure older adults’ healthy and inclusive aging.

The effect of age on eHealth literacy varies among different age groups (Table 4). The skill increases with age in young and middle-aged adults and declines with age in older adults [32,33]. Younger older adults are more likely to have better eHealth literacy than those who are older. This may be due to an age-related decline in physiological and cognitive ability to access eHealth information. A qualitative study stated that younger older adults adopt digital technology considering literacy, benefits, and support from relatives and families, while those who are older are more likely to adopt with support from friends and relatives [34]. Therefore, older adults are not homogenous, and improving eHealth literacy in older adults should consider age-specific interventions tailored to the needs of different older adults.

Previous studies have stated that economic status has a significant association with eHealth literacy, which is concurrent with this study [35]. Older adults with higher incomes are more likely to adopt digital devices and have more autonomy in internet use [36]. Lower-income older adults may face poor access to digital technology, leading to skill disparity. As South Korea ranked highest in old age poverty among Organization for Economic Co-operation and Development countries [37], the relationship between financial security, social support for older adults, and eHealth literacy needs in-depth investigations to prevent the potential loss of the silver economy in a digitalized society.

Regarding other socioeconomic factors, there is inconsistency in the relationship between sex and eHealth literacy, and this study did not find any significant differences among different sex (Table 4). Level of education is a significant predictor of eHealth literacy in several studies [33,38]. However, this study did not find favorable relationships between education level and eHealth literacy, except that those with a high school education or above were more likely to have higher eHealth literacy in univariate analysis (Table 4).

Internet and social media use had a strong association with eHealth literacy. The frequency and time spent on the internet lead to different levels of eHealth literacy among older people, and those spending 3 hours or more have 35 times higher eHealth literacy than nonusers (Table 4). The internet penetration of older people is around 90% of the general population [39] in South Korea, which means that Korean older adults have favorable environmental access to the internet, and they are more likely to adopt digital devices [40]. In addition, having confidence in using the internet promotes the use of the internet for seeking health information and thus improves eHealth usability [41]. Therefore, comprehensive internet coverage serving as a foundation, boosting older adults’ self-efficacy with digital technology and comfortability with eHealth skills, is a promising way to narrow the usability gap.

The eHEALS was developed before the widespread use of social media, and there were few studies regarding the validity of eHEALS on social media use. This study showed that social media users had 3.97 times higher eHealth literacy than nonusers (Table 4), which is concurrent with Tennant et al’s [42] findings. The diffusion of social media into people’s daily lives has become a channel to distribute health information and promote health, especially during the COVID-19 pandemic. South Korea and China have effectively used social media for disease notification, updating health information, and promoting preventive behaviors [43,44]. People who use social media frequently are more likely to keep abreast with updated health information, have better eHealth literacy, and be able to adopt healthy behaviors.

This study also found that eHealth literacy significantly impacts the self-perception of health (Table 6). Better levels of eHealth literacy are related to better-perceived health through access to quality health information and prompt and adequate health-related decisions [45,46]. Healthy behavior, such as participation in annual medical checkups, is not associated with eHealth literacy in this study population. The study population’s participation in annual medical checkups is 90% (Table 2), higher than the national level of 80.3% in the group older than 40 years [47]. Due to the well-established health screening programs and high participation, this study could not find a significant impact of eHealth literacy on participation in health screening programs.

In addition, this study found that eHealth literacy influences digital technology use for health and care purposes (Table 6). People with better eHealth literacy can navigate the required information correctly, whereas people with poor eHealth literacy skills lack eHealth self-efficacy and pose a barrier to the adoption of eHealth services. Moreover, a higher eHealth literacy level increases the positive impact on perceived usefulness and ease of use in technology and facilitates the adoption of digital health technology [45]. Recently, a municipality in South Korea has introduced an artificial intelligence–featured call to prevent social isolation in the older population [46]. Such development affirmed that digital technology would narrow the unmet needs of health care and long-term care services in the future, provided that the users have the proper knowledge and self-efficacy in eHealth. Therefore, promoting eHealth literacy for the older population is fundamental in expanding eHealth services and eliminating the gray digital divide.

### Limitations

Due to the demographics and aging rate of the sample population and the location of the study site, generalizing the findings may underestimate internet use in the older Korean population. In addition, eHEALS measures perceived eHealth skills rather than the actual performance of using digital technology. The possible gap between perceived skill and actual application of eHealth literacy could not be excluded.

### Conclusions

eHealth literacy is an essential skill in the rapidly digitalizing world. It is important to learn about the factors associated with eHealth literacy in community-dwelling Korean older adults and the impact of eHealth literacy on health-related outcomes and digital technology use for health purposes. With the application of the results from this study, interventions to improve the eHealth literacy skills of older adults can be tailored to high-risk populations and narrow the gap in the usability of eHealth services by older adults.

## Abbreviations

aOR: adjusted odds ratio
eHEALS: eHealth Literacy Scale
